# MAP3K1: A Multifunctional Kinase at the Crossroads of Cancer Progression and Tumor Suppression

**DOI:** 10.3390/cells15070604

**Published:** 2026-03-28

**Authors:** Lelisse T. Umeta, Amarnath Natarajan

**Affiliations:** 1 Eppley Institute for Cancer Research, University of Nebraska Medical Center, Omaha, NE 68198, USA; 2 Division of Pharmaceutical Sciences, James L. Winkle, College of Pharmacy, University of Cincinnati, Cincinnati, OH 45267, USA

**Keywords:** MAP3K1, kinase, E3 ligase, signaling, oncogene, tumor suppressor

## Abstract

Mitogen-activated protein kinase kinase kinase 1 (MAP3K1) possesses dual enzymatic functions, i.e., kinase and E3 ubiquitin ligase activities, orchestrating proliferation, survival, apoptosis, DNA damage response, and immune modulation. Recent genomic and mechanistic studies have revealed MAP3K1’s paradoxical, context-dependent roles as both an oncogene and a tumor suppressor. We discuss MAP3K1’s multidomain architecture, featuring an N-terminal RING and PHD domain (E3 ligase activity), a TOG domain (microtubule dynamics), and a C-terminal kinase domain, enabling the integration of c-jun N-terminal kinase (JNK), p38 mitogen-activated protein kinase (p38 MAPK), extracellular signal-regulated kinase (ERK), and nuclear factor kappa B (NF-κB) signaling pathways. MAP3K1 functions as a molecular switch balancing survival and apoptosis, with caspase-3 cleavage at Asp878 activating pro-apoptotic JNK/p38 signaling. Genomic analyses across >35 cancer types reveal MAP3K1 alterations at frequencies of <1–14%, highest in breast and endometrial cancers. These alterations show tissue specificity: loss-of-function mutations predominate in hormone receptor-positive breast cancer with a favorable prognosis, whereas gain-of-function mutations in melanoma activate oncogenic ERK signaling. MAP3K1 mutations predict response to mitogen-activated protein kinase kinase (MEK) and phosphoinositide 3-kinase (PI3K) inhibitors, with mutant cancers showing higher MEK inhibitor response than wild-type tumors. Despite substantial progress, critical gaps remain regarding MAP3K1’s E3 ligase substrates, context-dependent activity determinants, and therapeutic strategies. Addressing these through inhibitor development, biomarker validation, and mechanistic studies will accelerate potential clinical translation of MAP3K1 biology.

## 1. Introduction

Mitogen-activated protein kinase (MAPK) signaling is an evolutionarily conserved signaling network that plays critical roles in both normal physiology and disease states [1,2,3]. The canonical MAPK pathway comprises a 3-step cascade of serine/threonine and tyrosine kinases that are activated by diverse stimuli, triggering phosphorylation cascades involving combinations of 24 MAP3Ks, 7 MAP2Ks, and 14 MAPKs to regulate cell proliferation, survival, motility, stress response, and apoptosis [1]. Consequently, dysregulation of MAPK pathway proteins rewires signaling cascades to facilitate cancer initiation and progression. This made proteins in the MAPK signaling network attractive therapeutic targets for cancer, with 10 FDA-approved monotherapies, four combination therapies targeting components of MAPK signaling, and several more under clinical investigation [2,3,4,5]. However, the efficacy of these drugs has been challenged by emerging resistance arising from crosstalk among parallel pathways, feedback loops, and feedforward circuits [6,7,8].

Among MAPK cascade components, Mitogen-Activated Protein Kinase Kinase Kinase (MAP3K1, also known as MEKK1) stands out for its unique structural domains and functional properties. Recent large-scale genomic studies have identified MAP3K1 as one of the most frequently mutated genes in luminal A breast cancer, with a significant number of mutations observed in other cancers, such as gastric cancer [8,9]. MAP3K1 is a serine-threonine kinase with multifaceted roles in cellular signaling [1]. MAP3K1 has a kinase domain in its C-terminal region, identified by sequence homology with the kinases Sterile 11 (Ste11) and Bypass Ras (Byr) in the yeast pheromone response pathway [10,11]. Early studies demonstrated that MAP3K1 is activated by tumor necrosis factor-alpha (TNF-α), leading to TNF receptor (TNFR) activation, which subsequently binds to and phosphorylates MAP2Ks, such as MAP2K4, which, in turn, activates the JNK and p38 signaling pathways [12,13,14,15]. Later studies showed that MAP3K1 is composed of a large N-terminal region with a Plant Homeodomain (PHD) domain, which harbors two zinc finger domains (SWI2/SNF2 and MuDR (SWIM)- and Really Interesting New Gene (RING)-type). Recent studies have identified multiple domains with significant signaling consequences, such as the tumor overexpressed gene (TOG) domain, which binds to curved tubulin heterodimers [16]. To date, MAP3K1 is the only MAPK member with two domains with enzymatic activity: a kinase domain that phosphorylates substrates and an E3 ligase domain that ubiquitinates substrates [17]. MAP3K1 also functions as a scaffolding protein with binding sites for members of the MAPK signaling, such as the Rapidly Accelerated Fibrosarcoma (RAF), Mitogen-Activated Protein Kinase (MEK), Extracellular signal-regulated kinase (ERK), and C-jun N-terminal kinases (JNK) [18,19,20]. The signaling versatility of MAP3K1 is highlighted by its context-dependent regulation of ERK, p38, and JNK signaling to control cancer cell proliferation, survival, migration, invasion, and apoptosis, establishing its role in cell fate determination [21,22,23,24,25,26]. In addition to its role in regulating MAPK signaling, MAP3K1 activates NF-κB signaling and, in specific contexts, regulates Phosphoinositide 3-kinase (PI3K) and Protein kinase B (AKT) signaling [27] in cancer. In this review, we integrate genetic, molecular, and functional perspectives to examine how MAP3K1’s dual enzymatic activities orchestrate context-dependent oncogenic and tumor suppressor functions. We further discuss how MAP3K1 biology informs therapeutic strategies and biomarker development.

## 2. Domain Structure and Function of MAP3K1

**Figure 1:**
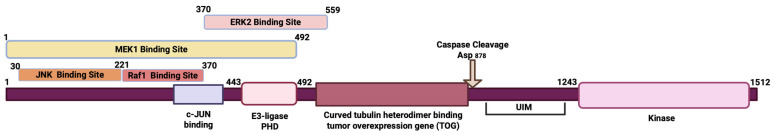
Domain architecture of MAP3K1. The N-terminal region (amino acids 1–1242) contains the SWIM zinc finger domain (aa 228–266), PHD/RING E3 ubiquitin ligase domain (aa 443–492), and TOG domain (aa 548–867) involved in tubulin binding and microtubule dynamics. The C-terminal region harbors the ubiquitin-interacting motif (UIM) and serine/threonine kinase domain (aa 1243–1508). Key regulatory sites are indicated: the Caspase-3 cleavage site at DTVD878 and critical autophosphorylation sites (Thr1281, Thr1393). Binding regions for upstream activators (Raf1) and downstream substrates (c-jun, MEK, ERK, JNK) are also marked. Created with BioRender.com.

To understand MAP3K1’s multifaceted roles in cancer, we first examine its modular domain architecture and regulatory mechanisms. The *MAP3K1* gene is located on Chromosome 5q11.2. MAP3K1 is composed of 1512 amino acids, with residues 1243–1508 harboring the C-terminal kinase domain and ubiquitin-interacting motif (UIM) predicted immediately upstream of the kinase domain. Using computational sequence alignment and experimentally determined spacing between conserved metal-chelating cysteine and histidine residues, residues 228–266 were found to contain a SWI2/SNF2 and MuSK (SWIM) domain, and residues 443–492 to contain the Plant Homeodomain/Really Interesting New Gene domain (PHD/RING) zinc finger domain. Additionally, MAP3K1 possesses a TOG curved tubulin-binding domain at residues 548–867, and a Caspase-3 cleavage site at residue Asp-Thr-Val-Asp-878 (DTVD878) [16,17,28] (Figure 1).

These distinct structural domains enable MAP3K1 to integrate and regulate multiple signaling cascades. Activation of MAP3K1 is regulated by phosphorylation of Thr1281 and Thr1393. While the exact mechanism of activation remains unclear, Germinal Center Kinase (GCK) and TNF receptor-associated factor 2 (TRAF2) are common upstream activators of MAP3K1. GCK activates MAP3K1 through GCK’s non-catalytic C-terminal domain by promoting MAP3K1 oligomerization, which triggers MAP3K1 autophosphorylation at T1381 and T1393. The TRAF2 RING domain is essential for optimal MAP3K1 activation, providing the first evidence of direct in vitro activation of MAP3K1 and establishing a model where upstream activators induce MAP3K1 oligomerization to trigger its activation through autophosphorylation [29,30]. MAP3K1 can also be activated through autophosphorylation. In addition, MAP3K1 also undergoes auto-ubiquitination without degradation, resulting in inactivation of its kinase activity [31]. MAP3K1 is also phosphorylated by several serine/threonine kinases, such as the hematopoietic progenitor kinase 1 (HPK1), Nck-interacting kinase (NIK), c-Abl, GCK-like kinase (GLK), Protein Kinase C (PKCβ), and Glycogen synthase kinase-3 beta (GSK3β), and at various residues in the N terminus to regulate downstream signaling [32,33,34].

MAP3K1 plays an important role in the cellular stress response, which is perhaps its most studied function. In response to hyperosmotic stress, MAL and Related proteins for Vesicle trafficking and membrane Link (MARVEL) domain-containing protein 3 (MarvelD3), a transmembrane tight junction sensor, complexes with MAP3K1 and translocates MAP3K1 to the cell membrane to activate the cellular stress response [35]. The membrane-bound full-length MAP3K1 (196 kDa) is cleaved by Caspase 3, yielding an activated, 91 kDa C-terminal kinase whose cytosolic localization is required for the induction of apoptosis; either caspase cleavage site mutation or kinase inactivation attenuates MAP3K1-mediated apoptosis [36]. This proteolytic switch exemplifies how cellular context, in this case, apoptotic signals, can redirect MAP3K1 from pro-survival to pro-apoptotic signaling.

MAP3K1 differentially activates JNK, p38, ERK, and NF-κB pathways depending on cellular context and stimulus type. Although MAP3K1 phosphorylates MEK in vitro and activates the MEK/ERK signaling pathway [20], it primarily regulates the JNK/p38 and NF-κB signaling pathways. Upon proinflammatory cytokine stimulation, MAP3K1 phosphorylates serine residues in the activation loop of IKKs (177 and 181 on IKKβ and 176 and 180 on IKKα), thereby activating the IKK complex and initiating subsequent NF-κB signaling [13,30,37,38,39]. MAP3K1’s E3 ligase activity mediates K48-linked ubiquitination of c-jun and ERK1/2 for proteasomal degradation in response to genotoxic and osmotic stress [19,28,40,41,42,43]. A comprehensive study using a knock-in mouse model with an inactive MAP3K1 PHD motif identified 82 potential substrates of MAP3K1’s E3 ligase activity, including K63-linked ubiquitination of TGFβ-activated kinase 1 (TAK1)-binding protein 1 (TAB1) for TAK1 activation [44]. However, systematic functional validation of these putative substrates, including determination of ubiquitin linkage type, assessment of functional consequences, and characterization of context-dependency, has yet to be fully realized. MAP3K1’s broad influence across multiple pathways discussed above highlights its remarkable context-dependent function, acting as a molecular switch that can induce cellular apoptosis or promote cell survival.

## 3. MAP3K1 in Cancer Signaling

### 3.1. JNK/P38 Signaling

**Figure 2:**
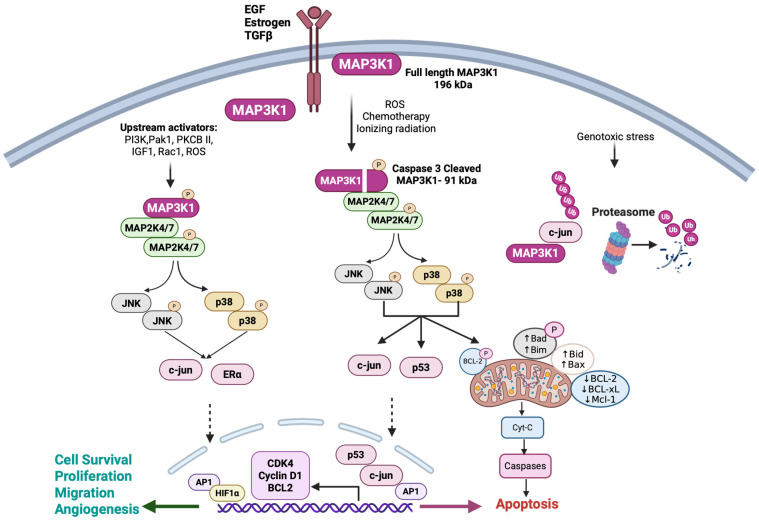
Context-dependent regulation of the JNK/p38 signaling. Full-length MAP3K1 (196 kDa, left) responds to growth factor and stress signals, activating MAP2K4 to activate JNK/p38 pathways and MAP2K7 to activate the JNK pathway to drive transcription (c-jun, ERα, AP-1, HIF-1α), thereby promoting survival, proliferation, migration, and angiogenesis. Apoptotic stimuli trigger caspase-3 cleavage at Asp878, generating a 91 kDa C-terminal fragment that activates JNK/p38 with altered substrate preference for pro-apoptotic targets, leading to mitochondrial BCL-2 family dysregulation, cytochrome c release, caspase activation, and apoptosis. Under genotoxic stress, MAP3K1’s E3 ligase activity ubiquitinates c-jun for proteasomal degradation, regulating c-jun protein levels. Phosphorylation is shown as a circled “P” and ubiquitination is shown as a circled “Ub”. Created with BioRender.com.

MAP3K1 activates JNK and p38 signaling across multiple cancer types, exerting context-dependent effects (Figure 2). MAP3K1 is an upstream kinase of JNK and p38, which, in turn, phosphorylate and activate downstream signaling molecules to promote nuclear translocation and activation of critical transcription factors such as activator protein 1 (AP-1). As a result, this signaling axis regulates various phenotypes that are specific to the stimuli and cancer type. In glioblastoma, MAP3K1 levels are higher in peripheral infiltrating regions than in the tumor core, where it activates the JNK/c-jun pathway to promote cancer cell proliferation and invasion; these effects are reversed by genetic knockout of MAP3K1 or by JNK inhibition [45]. In glioma, PI3K and P21-activated kinase 1 (Pak-1) signaling regulate the MAP3K1-JNK axis by sustaining MAP3K1 phosphorylation. This prevents MAP3K1-JNK interaction and keeps activated JNK in the cytoplasm, which is associated with increased glioma cell migration. However, when Pak-1 is inhibited, MAP3K1 associates with JNK to facilitate JNK nuclear translocation, resulting in reduced cell motility [46]. On the other hand, in papillary thyroid carcinoma, downregulation of MAP3K1 inhibited downstream JNK/c-jun signaling, which is associated with cancer cell invasion and migration by regulating matrix metalloproteases (MMPs) and vimentin. These phenotypes were reversed when MAP3K1 was knocked out in a papillary thyroid carcinoma model [47]. Similarly, JNK-c-jun signaling activation increased Cyclin D1 and Cyclin-dependent kinase 4 (CDK4) expression, facilitating cell proliferation, and increased B-cell lymphoma 2 (Bcl-2) and decreased Bcl-2-associated X protein (Bax) levels, promoting cancer cell survival. In hepatocellular carcinoma, the MAP3K1-JNK-AP-1 pathway interacts with hypoxia-inducible factor-1 (HIF-1) to maximize vascular endothelial growth factor (VEGF) gene expression during hypoxia; inhibition of either pathway significantly suppresses hypoxia-induced VEGF expression [48]. In breast cancer, upstream Ras-related C3 botulinum toxin substrate 1 (Rac1) signaling activates MAP3K1, which in turn activates p38 and JNK signaling, leading to direct phosphorylation and activation of estrogen receptor alpha (ERα) by p38 and its indirect activation through JNK. In the uterus, MAP3K1 significantly enhances the agonistic activity of 4-hydroxytamoxifen to match 17β-estradiol levels and blocks its antagonistic properties, suggesting there are MAP3K1-mediated, tissue-specific effects of tamoxifen [49]. In addition, MAP3K1 mediates the convergence of estrogen and insulin-like growth factor 1 (IGF-1) signaling by activating the MAP2K7/JNK cascade to increase c-jun expression and drive cancer cell growth by hijacking the reactive oxygen species (ROS) signaling in breast cancer [50]. MAP3K1 functions as a redox-sensitive kinase, with protein kinase C beta II (PKCβII) activating MAP3K1 through both direct interaction and a TPA-induced ROS-dependent mechanism, an event linked to the proliferative response [51].

On the other hand, in a context-dependent manner, MAP3K1 functions as a tumor suppressor that regulates the cellular apoptotic response. A recent mechanistic study showed that MAP3K1 loss-of-function mutations drive breast cancer progression, suggesting a tumor suppressor function. Loss of MAP3K1 or MAP2K4 reduced JNK2 phosphorylation, which impairs p53 phosphorylation at Ser15 and mediates the upregulation of FOSL1, a transcription factor that promotes tumor proliferation and metastasis. Importantly, MAP3K1/MAP2K4 mutations were mutually exclusive with *TP53* alterations in breast cancer, particularly in estrogen receptor-positive (ER+) tumors, suggesting that these mutations provide an alternative mechanism for p53 pathway disruption [52]. Under ionizing radiation (IR), MAP3K1 mediates the sustained phosphorylation and activation of JNK and p38 in radiation-sensitive lung cancer cells, resulting in Bak activation and mitochondrial depolarization, leading to apoptosis [53]. In prostate cancer cells, DHT-induced androgen receptor (AR) activity promotes MAP3K1-mediated sustained JNK activation, resulting in apoptosis rather than cell survival [54]. The C-terminal fragment of MAP3K1 has been implicated in driving apoptotic signaling across multiple cancer types. Treatments including paclitaxel, cisplatin, and vitamin D3 induce caspase-3-mediated cleavage of MAP3K1 at Asp878, generating a constitutively active 91 kDa C-terminal fragment that retains full kinase activity. The resulting C-terminal cytoplasmic MAP3K1 fragment preferentially activates JNK and p38 signaling pathways over ERK signaling, suggesting altered substrate selectivity. However, systematic substrate profiling comparing specific phosphorylation site preferences between full-length and truncated MAP3K1 has not been comprehensively reported. Nonetheless, the activation of the JNK/p38 signaling, which directly and indirectly regulates mitochondrial proteins such as BCL-2 and BAK, tips the balance toward pro-apoptotic signaling [36,54,55,56,57]. Beyond cell fate determination, MAP3K1 also regulates cellular differentiation processes in various cell types. Activated Ras and phosphorylated PKCδ at tyrosine 311 trigger MAP3K1 activation and AP-1 transcription factors to regulate keratinocyte differentiation [58]. In myeloid leukemia, tissue plasminogen activator (TPA) stimulation results in PKCβII-mediated phosphorylation of both full-length and cleaved MAP3K1 [59]. Although earlier studies showed that the PHD domain of MAP3K1, under osmotic and genotoxin-induced stress conditions, regulates ERK and c-jun function through proteasomal degradation [41,43], subsequent studies showed that this role also extends to other members of the MAPK signaling pathways. The MAP3K1 PHD motif, along with ubiquitin-conjugating enzyme E2 N (UBE2N) and UBE2 variant 1 (UBE2V1), transfers Lys63-linked polyubiquitin onto TAB1, which is essential for JNK and p38 activation in response to cytokines like transforming growth factor beta (TGF-β) and EGF [60]. These context-dependent outcomes likely reflect stimulus-specific activation of distinct JNK/p38 pools, potentially driven by differential subcellular localization and scaffolding capacity between full-length MAP3K1 and its cytoplasmic 91 kDa fragment, though the molecular basis for this compartmentalization remains to be investigated.

### 3.2. MEK-ERK Signaling

**Figure 3:**
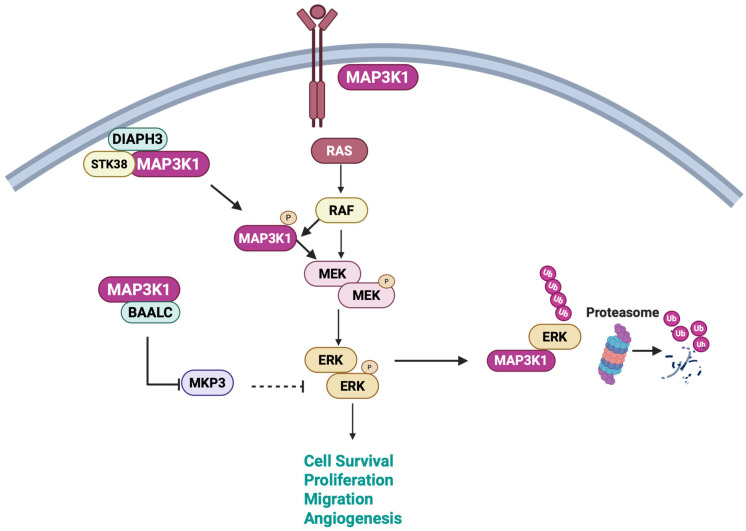
Dual regulation of ERK signaling by MAP3K1. MAP3K1 both activates and inhibits ERK signaling through distinct mechanisms. Through kinase activity, MAP3K1 phosphorylates RAF in the RAS-RAF-MEK-ERK cascade, using scaffold proteins DIAPH3 and STK38 to promote survival and proliferation. MAP3K1 complexes with adapter BAALC to inhibit the ERK phosphatase MKP3 (dashed line). Through E3 ligase activity, MAP3K1 ubiquitinates ERK for proteasomal degradation in response to specific cellular stress signals. Phosphorylation is shown as a circled “P” and ubiquitination is shown as a circled “Ub”. Created with BioRender.com.

MAP3K1 regulates the MEK-ERK pathway (Figure 3) through a complex multifunctional mechanism. In normal cells, upstream kinases such as serine/threonine kinase 38 (STK38) associate with MAP3K1 to negatively regulate MAP3K1 and inhibit the activation of the MEK-ERK cascade. In lung adenocarcinoma, upregulation of diaphanous-related formin 3 (DIAPH3), a protein involved in actin and microtubule organization, associates with STK38 to disrupt the MAP3K1-STK38 interaction, thereby activating MEK-ERK signaling to drive cell proliferation and tumor growth [61]. In acute myeloid leukemia, upon phorbol myristate acetate (PMA) stimulation, MAP3K1 forms complexes with brain and acute leukemia cytoplasmic (BAALC) and blocks mitogen-activated protein kinase phosphatase 3 (MKP3)-mediated ERK deactivation, which drives cell proliferation and chemoresistance. This model supports the observed efficacy of MEK inhibitors in BAALC-high leukemia cells [62]. MAP3K1 phosphorylates progesterone receptor (PR) at serine 294. This phosphorylation first hyperactivates PR transcriptional activity, then subsequently targets PR for proteasomal degradation, creating a negative feedback loop. The study demonstrated that PR hyperactivation and degradation are mechanistically coupled: inhibiting S294 phosphorylation (via MAPK inhibitors or S294A mutation) or blocking proteasomal degradation (via proteasome inhibitors) abolishes MAP3K1-induced transcriptional hyperactivity. The coupling between hyperactivation and degradation represents a temporal regulatory mechanism: enhanced transcriptional output is limited by accelerated receptor turnover, where, without S294 phosphorylation-triggered degradation, PR remains in a non-hyperactive conformation [63]. Furthermore, MAP3K1 regulates ERK levels through ubiquitination and proteasomal degradation under osmotic stress induced by sorbitol treatment [43].

### 3.3. NF-κB

**Figure 4:**
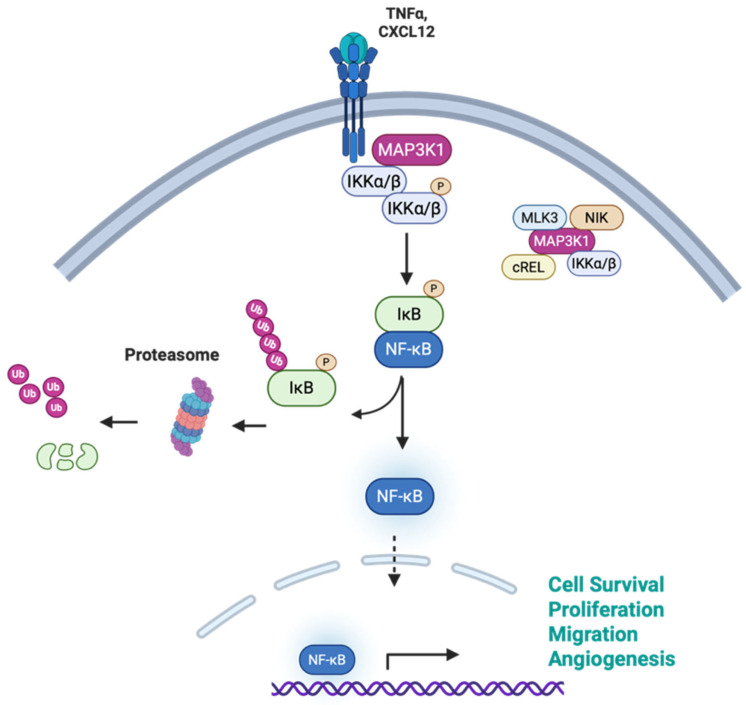
MAP3K1 activates canonical NF-κB signaling. In response to TNFα and CXCL12 stimulation, MAP3K1 phosphorylates IKKα/β, which in turn phosphorylates inhibitor of kappa B(IκB), targeting it for K48-Linked Ubiquitination and proteasomal degradation. IκB degradation releases NF-κB, which translocates to the nucleus and activates genes involved in survival, proliferation, migration, and angiogenesis. MAP3K1 also interacts with other pathway activators, such as NIK, MLK3, and cREL, and phosphorylates IKKα/β to activate the NF-κB signaling. Created with BioRender.com.

Another common oncogenic signaling pathway regulated by MAP3K1 is NF-κB signaling (Figure 4). MAP3K1 activates both IkappaB kinase (IKKα and IKKβ) kinases within the heterotrimeric IKK signalosome complex. While the mechanistic basis for MAP3K1-mediated activation remains to be fully elucidated, published reports indicate that activation is sequential. MAP3K1 phosphorylates and activates IKKα, which, in turn, results in IKKβ phosphorylation, similar to and in cooperation with other IKK activators such as mixed-lineage protein kinase 3 (MLK3) and NF-κB-inducing kinase (NIK) [64,65,66]. MAP3K1-mediated NF-κB regulation has been implicated in various cancers. In chronic myelogenous leukemia (CML), driven by the BCR-ABL fusion oncoprotein, MAP3K1 is upregulated and phosphorylated downstream of BCR-ABL signaling, leading to activation of NF-κB signaling and promoting cancer cell survival. CML cells expressing dominant negative MAP3K1 displayed reduced NF-κB activity and were sensitive to apoptosis under genotoxic stress [67]. In invasive breast cancer, where Annexin A1 is overexpressed, Ras homology family member A (RhoA) activates the MAP3K1-NF-κB pathway in response to CXC motif chemokine ligand 12 (CXCL12) stimulation, to promote the migration of invasive breast cancer cells [68]. In normal pancreatic cells, MAP3K1-mediated IKKβ activation is transient in response to TNF-α stimulation; however, in pancreatic cancer cells, this activation is sustained, driving persistent NF-κB signaling. Specifically, MAP3K1 phosphorylates IKKβ at serines 177 and 181 in its activation loop to trigger the nuclear translocation of NF-κB transcription factors and to promote pancreatic cancer cell survival, proliferation, and metastasis. Consistently, high *MAP3K1* mRNA expression correlates with poor survival in pancreatic cancer patients [69].

## 4. Context-Dependent Role of MAP3K1 in Cell Fate Determination

**Figure 5:**
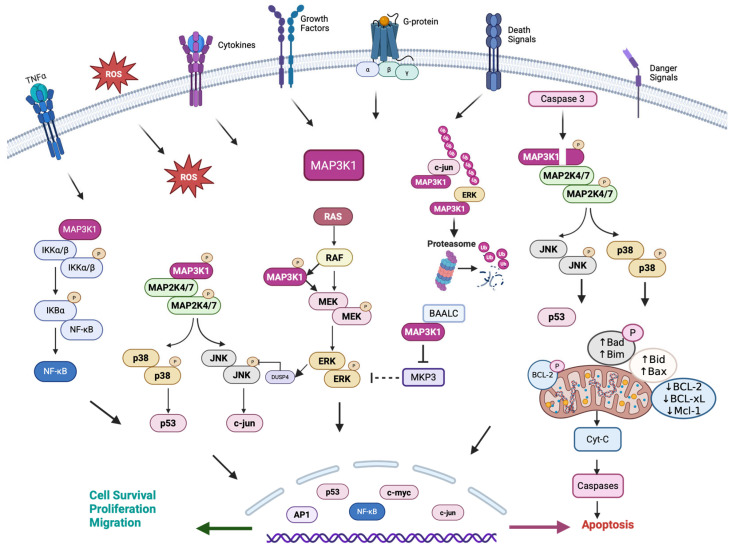
MAP3K1 signaling networks and context-dependent cellular outcomes. MAP3K1 integrates diverse upstream signals (TNF, ROS, cytokines, growth factors, GPCRs, death receptors, danger signals) to activate four major downstream pathways: NF-κB, ERK, JNK, and p38. MAP3K1 regulates transcription factors (p53, AP1, NF-κB, c-myc, c-jun), which control cell survival, proliferation, and migration. Caspase cleavage of MAP3K1 generates a C-terminal fragment that preferentially activates the JNK/p38 pathways, promoting apoptosis by regulating BCL-2 family proteins, mitochondrial dysfunction, cytochrome c release, and caspase activation. MAP3K1 marks c-jun and ERK for degradation under cellular stress signaling pathways. Phosphorylation is shown as a circled “P” and ubiquitination is shown as a circled “Ub”. Created with BioRender.com.

One of the most intriguing aspects of MAP3K1 biology is its contradictory roles across different cancer types. MAP3K1’s role in determining cell fate exhibits remarkable context-dependency across different signaling environments (Figure 5). This paradox has significant implications for therapeutic targeting and patient stratification. In human bronchial epithelial cells, MAP3K1 functions downstream of TNF-α to activate the AP-1 and NF-κB transcription factors. Kinase-inactive MAP3K1 impairs, while constitutively active MAP3K1 enhances, the expression of genes linked to inflammation, proliferation, and survival [70]. On the other hand, TNF-α binding to TNFR1 induces receptor clustering, facilitating MAP3K1 association and activation of the JNK/p38 signaling pathways, leading, in a context-dependent manner, to either cellular apoptosis or cell survival [71,72,73,74]. For example, TNF-α stimulation activates the MAP3K1-JNK signaling axis, leading to JNK-mediated phosphorylation of tristetraprolin (TTP) at serine residues. This phosphorylation primes TTP for TRAF2-mediated K48-linked ubiquitination and proteasomal degradation (hypermodification). While unmodified TTP destabilizes pro-inflammatory mRNAs, TTP hypermodification relieves this brake, promoting sustained inflammatory signaling through NF-κB and JNK pathways [75]. Additionally, MAP3K1 significantly increases c-myc protein stability by extending its half-life independently of transcription downstream of TNF-α [76].

MAP3K1 exhibits tumor-suppressive functions in specific cancer contexts. In Hedgehog-driven medulloblastoma, MAP3K1 physically associates with the zinc finger protein GLI1 (GLI1) and phosphorylates its C-terminal region, promoting 14-3-3 protein binding and inhibiting GLI1’s transcriptional activity. Overexpression or pharmacological activation of GLI1 significantly reduces GLI1 activity, tumor cell proliferation, and viability [77]. In prostate cancer, miRNA-627 significantly upregulates MAP3K1 and other tumor suppressor genes such as the receptor-type tyrosine-protein phosphatase kappa (PTPRK) and steroid receptor RNA activator 1 (SRA1), inhibiting cancer cell proliferation, cell cycle progression, migration, and promoting apoptosis [78]. MAP3K1 functions as a key negative regulator of telomerase RNA (hTR) gene expression via JNK signaling; constitutively active MAP3K1 significantly represses hTR promoter activity. JNK inhibition reverses this effect and increases endogenous hTR levels by modulating transcription factor dynamics, enhancing repressor transcription factor Sp3 binding, and reducing activator transcription factor Sp1’s influence [79]. Etoposide treatment activates proapoptotic signaling through MAP3K1, which plays a critical role in death receptor-mediated apoptosis by activating the p65 subunit of NF-κB, which, in the nucleus, increases DR4 transcription and links DNA damage responses to death receptor expression. Through this mechanism, MAP3K1 sensitizes cancer cells to TNF-related apoptosis-inducing ligand (TRAIL)-induced apoptosis and is rescued by the MAP3K1 kinase mutation [80]. Etoposide treatment upregulates death receptors, which, when bound by TRAIL, enhance apoptotic signaling by activating both the MAP3K1-NF-κB pathway [81] and the MAP3K1-JNK/p38 pathway [82,83], providing essential insights into how MAPK signaling cascades are regulated during death receptor-mediated apoptosis. In prostate cancer cells, constitutively active MAP3K1 promotes AR-mediated gene transcription and triggers apoptosis specifically in cells with functional AR pathways through a caspase-dependent but JNK-independent mechanism [84]. MAP3K1 also interacts with Axin through its MAP3K1-interacting domain (MID), distinct from the domains essential for Wnt signaling. Dimerization/oligomerization of MAP3K1 and Axin through its C-terminus mediates JNK activation, and Axin mutants lacking the MID had a dominant-negative effect on wild-type Axin [85]. Consequently, because of its role in regulating signaling pathways that mediate cell survival or apoptosis, MAP3K1 exhibits opposing functions across cancer types. For example, in luminal breast cancer, MAP3K1 predominantly functions as a tumor suppressor through loss-of-function mutations, which are associated with a favorable prognosis (HR = 0.62, *p* < 0.001) [86,87]. Conversely, in melanoma, activating translocations produce truncated MAP3K1 proteins with constitutive kinase activity, driving oncogenic ERK signaling [88,89]. MAP3K1 is significantly upregulated in gastric cancer, where it drives proliferation and correlates with poor survival, versus being downregulated in anaplastic thyroid carcinoma progression from papillary thyroid carcinoma. Both gastric and thyroid cancers show epigenetic dysregulation, with promoter hypomethylation driving gastric cancer upregulation and miRNA-mediated suppression facilitating thyroid cancer progression [90,91].

MAP3K1’s core signaling outputs include the JNK/p38, MEK-ERK, and NF-κB pathways, with paradoxical roles in cell survival and death, indicating that MAP3K1 is a molecular cell-fate switch. We summarize how these signaling networks converge to drive specific cancer hallmarks. The multidomain architecture of MAP3K1 enables it to orchestrate complex cellular processes beyond the activation of classical cytoplasmic pathways. Through coordinated action of its kinase activity, E3 ligase function, and scaffolding capabilities, MAP3K1 integrates signals that determine critical cancer phenotypes, including genomic stability, cellular motility, and immune modulation. This signaling versatility exhibits remarkable context-dependence: MAP3K1 responds differentially to upstream stimuli (growth factors, cytokines, osmotic stress, genotoxic signals), and its cleavage state (full-length versus caspase-3-cleaved C-terminal fragment) fundamentally alters substrate selectivity and pathway activation. As we explore in the following sections, additional determinants, including tumor type, mutation class, and co-mutation landscape, further shape whether MAP3K1 functions as a tumor suppressor or oncogene, with critical implications across key hallmarks of cancer, thereby positioning MAP3K1 as a central node that coordinates multiple aspects of tumor biology.

## 5. MAP3K1 in DNA Damage Response and Transcriptional Regulation

**Figure 6:**
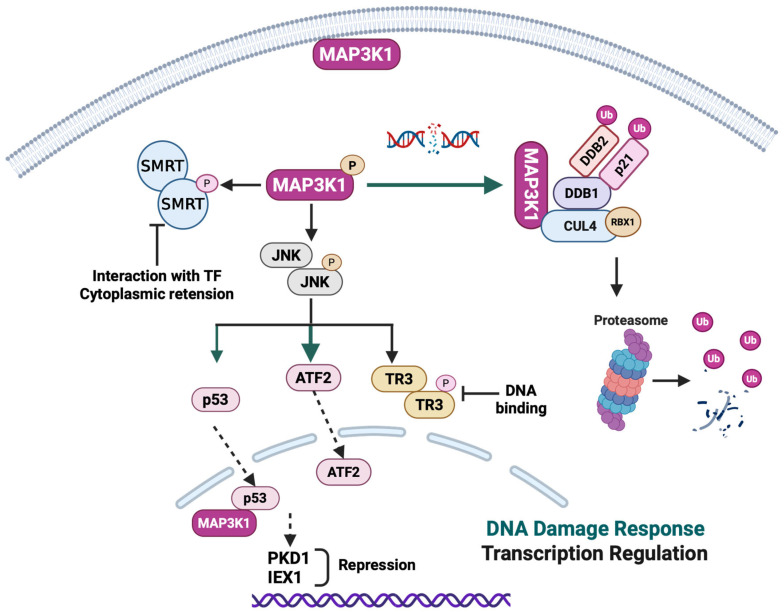
MAP3K1 regulates DNA damage response and transcription. MAP3K1 regulates DNA damage response and transcription through kinase and E3 ligase activities. Through JNK phosphorylation, MAP3K1 activates the transcription factors ATF2 and TR3, thereby regulating the expression of genes that respond to DNA damage. MAP3K1 phosphorylates SMRT, altering transcription factor interactions and cytoplasmic retention. MAP3K1 cooperates with p53 to repress PKD1 and IEX1 expression. As an E3 ligase, MAP3K1 complexes with DDB2, p53, DDB1, CUL4, and RBX1 to ubiquitinate and degrade DNA damage response proteins p21 and DDB2. Phosphorylation is shown as a circled “P” and ubiquitination is shown as a circled “Ub”. Created with BioRender.com.

MAP3K1 plays critical roles in nuclear processes, spanning both the DNA damage response and transcriptional regulation (Figure 6), through diverse mechanisms that extend beyond its canonical cytoplasmic MAPK signaling functions. In the DNA damage response signaling pathway, MAP3K1 activates the transcription factor Activating Transcription Factor 2 (ATF-2). In human neuroblastoma cells, MAP3K1 expression increased ATF-2 activity via the JNK pathway rather than the p38 MAPK pathway [92]. In addition, MAP3K1 is associated with increased topoisomerase IIa (TOP2A) promoter activity through its dual activation of the ERK and JNK pathways necessary to achieve a robust induction of TOP2A expression [93]. Through JNK signaling, MAP3K1 stabilizes and activates Tumor Protein p53 (p53) by prolonging its half-life. This stabilization is achieved through two complementary mechanisms. First, MAP3K1 promotes the dissociation of p53 from Mouse Double Minute 2 (MDM2), its primary negative regulator, thereby reducing MDM2-mediated ubiquitination and proteasomal degradation of p53. Second, the increased association of p53 with MAP3K1 facilitates p53 phosphorylation, which enhances its transcriptional activity and pro-apoptotic function [94].

Beyond its direct E3 ligase function, MAP3K1 serves as a novel constitutive interactor of the Cullin-RING Ligase 4 (CRL4) complex in the cytosol, functioning as a regulatory component in CRL4’s DNA damage response. The kinase activity of MAP3K1 triggers autoubiquitination of the CRL4 complex, leading to the formation of K48- and K63-linked ubiquitin chains, which are essential for the degradation of CRL4 substrates such as Cyclin-Dependent Kinase Inhibitor 1A (p21) and DNA Damage-Binding Protein 2 (DDB2) during DNA damage response signaling [95]. A recent phosphoproteomic analysis using an ATP-competitive MAP3K1 inhibitor identified significant changes in multiple phosphosites of nucleophosmin (NPM1), including T199, a phosphosite important for NPM1 nuclear localization to DNA damage sites [96]. Together, these studies highlight the role of distinct MAP3K1 domains working in concert to regulate DNA damage response.

MAP3K1’s role extends beyond kinase-mediated activation of transcription factors to direct transcriptional regulation through both kinase-dependent and independent mechanisms. Under oxidative stress (H_2_O_2_), PMA, and TNFα stimulation, MAP3K1 interacts with p53 and facilitates p53 nuclear translocation. At target gene promoters, including protein kinase D1 (PKD1) and immediate early response gene X-1 (IEX-1), MAP3K1 functions as a transcriptional co-repressor in a complex with DNA-bound p53. This forms a mutually dependent repressor complex: MAP3K1 requires p53 for promoter recruitment (as MAP3K1 lacks DNA-binding activity), while p53-mediated transcriptional repression requires MAP3K1 co-repressor activity [97].

Furthermore, the MAP3K1-JNK signal regulates cytomegalovirus (CMV) promoter activity through JNK binding to the proximal cyclic AMP-responsive element (CRE) binding site at position -166 in the promoter. Deletion of this CRE sequence or the use of JNK inhibitor abolished CAM’s activation of the CMV promoter, confirming the involvement of the MAP3K1-JNK pathway [98]. In lung cancer cells, MAP3K1 inhibits the mitogenic function of the nuclear receptor 4A1 (also known as TR3, NGFI-B, or Nur77) through JNK1 activation, which phosphorylates TR3 primarily on its N-terminus, thereby inhibiting DNA binding, suppressing transcriptional activity, and inhibiting TR3-induced cell proliferation [99]. MAP3K1 phosphorylates the silencing mediator of retinoid and thyroid hormone receptors (SMRT) corepressor, which not only disrupts SMRT’s ability to interact physically with its transcription factor partners but also leads to its redistribution from the nucleus to the cytoplasm and influences gene expression [100]. In colon carcinoma cells, MAP3K1 plays a critical role in the thymineless stress response by regulating NF-κB and AP-1 transcription factors, thereby upregulating Fas ligand (FasL) expression and inducing apoptosis. Dominant-negative MAP3K1 blocks AP-1 activation and reduces FasL expression, thereby enhancing clonogenic survival under thymidine-deficient conditions by dampening apoptosis [101]. This integration of DNA damage signaling and transcriptional control positions MAP3K1 as a key coordinator of nuclear responses to cellular stress, linking external stimuli to gene expression and, in turn, to cellular signaling and cell fate.

## 6. MAP3K1 in Cell Motility: Cell Invasion, Migration, and Metastasis

**Figure 7:**
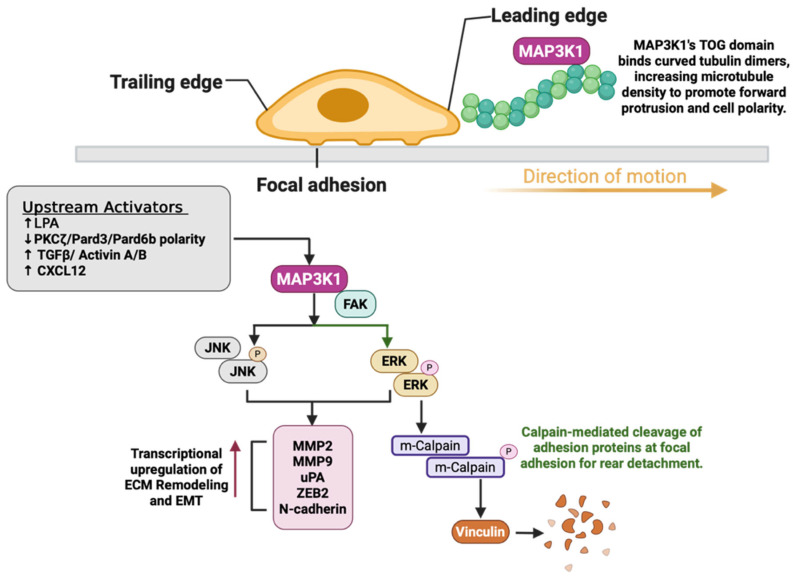
MAP3K1 coordinates multiple steps of the metastatic process from local invasion through distant colonization. MAP3K1 localizes to focal adhesions and forms complexes with FAK. It activates ERK1/2 at these sites, thereby triggering calpain-mediated cleavage of adhesion proteins (e.g., vinculin), enabling rear-end detachment during migration. The TOG domain binds tubulin dimers, regulating microtubule dynamics at the cell leading edge. MAP3K1 upregulates uPA expression via AP-1 activation, thereby promoting plasmin generation and matrix metalloproteinase (MMP) activation. MAP3K1 promotes epithelial–mesenchymal transition through JNK and ERK signaling, reducing E-cadherin and increasing N-cadherin, vimentin, ZEB2, and Snail expression. Phosphorylation is shown as a circled “P”. Created with BioRender.com.

A well-characterized function of MAP3K1 is its role in cell motility (Figure 7). MAP3K1 coordinates the metastatic cascade from the assembly of focal adhesions at the leading edge and their disassembly at the trailing edge, to extracellular matrix degradation, to epithelial–mesenchymal transition (EMT), ultimately enabling cancer cells to establish distant metastases. MAP3K1 plays a critical role in regulating cell motility across multiple cancer types, as evidenced by MAP3K1 knockout mice that exhibit epithelial sheet migration defects and an inability to close their eyelids [102]. Subsequent studies revealed that both MAP3K1 knockout and its kinase deficiency impair cell motility, with MAP3K1 kinase-deficient embryonic stem (ES) cells showing reduced serum and lysophosphatidic acid (LPA)-induced migration [14]. Epidermal keratinocytes from MAP3K1-deficient mice display reduced JNK1/2 activation, a mechanism by which keratinocytes activate actin stress fibers following TGF-β and Activin A/B stimulation, resulting in impaired cell motility [89,90]. MAP3K1-deficient mouse embryonic fibroblasts (MEF) and stem cells exhibit impaired adherence to cell culture plates and reduced migration toward serum, fibronectin, and growth factors [14,24]. At the same time, MAP3K1 overexpression promotes the formation of lamellipodia-like structures that facilitate movement [103].

The molecular mechanisms underlying MAP3K1’s regulation of cell motility involve multiple pathways and structural interactions. MAP3K1 localizes to focal adhesions in fibroblasts, forming complexes with focal adhesion kinase (FAK) upon growth factor stimulation. It activates ERK1/2 at focal adhesions, leading to calpain activation that cleaves focal adhesion proteins essential for rear-end detachment during migration. As a result, MAP3K1-deficient cells show reduced vinculin at focal adhesions, decreased MAPK1/3 phosphorylation in response to growth factors, and impaired calpain activation [24,104,105,106]. Additionally, MAP3K1 regulates urokinase-type plasminogen activator (uPA) expression by modulating AP-1 transcription factors, resulting in plasmin-mediated extracellular matrix degradation [25]. The recently reported TOG domain in MAP3K1 binds tubulin dimers. It preferentially binds curved tubulin heterodimers found in soluble tubulin and at microtubule polymerization/depolymerization sites. Disruption of this tubulin-binding interface decreases microtubule density at the leading edge of polarized cells. Furthermore, mutations in the TOG domain appear recurrently in patient-derived tumor samples, suggesting a potential selection advantage during cancer progression [16].

MAP3K1’s role in cancer metastasis has been established across multiple malignancies. In ovarian cancer, elevated LPA levels activate MAP3K1 through a Gi-RAS-dependent mechanism. This enhances the metastatic potential by regulating the redistribution of focal adhesion kinase to focal contacts. Consistently introducing a dominant-negative MAP3K1 inhibits ~80% of this migratory response [107]. In pancreatic adenocarcinoma, MAP3K1 expression significantly correlates with lymph node metastasis; all positive cases show MAP3K1 staining, and siRNA depletion of MAP3K1 dramatically inhibits invasion, migration, adhesion, MMP2 activity, and ERK1/2 phosphorylation [23]. In lung cancer, disruption of the PKCζ/Pard3/Pard6b polarity complex upregulates MAP3K1, triggering epithelial–mesenchymal transition (EMT) and enhanced invasiveness. Pard3 suppression alters genes regulating wound healing, motility, and apoptosis, with MAP3K1 being significantly upregulated at both the mRNA and protein levels [98]. In non-small cell lung cancer (NSCLC), downregulation of miR-770, which targets MAP3K1, enables MAP3K1 to promote macrophage polarization toward the tumor-promoting M2 phenotype. Exosomal transfer of miR-770 reduced phosphorylation of JNK, ERK1/2, and inhibited migration, invasion, and EMT, both in vivo and in vitro. Notably, these effects were reversed when MAP3K1 levels were restored [108]. Single-cell RNA-Seq analysis of glioblastoma (GBM) revealed high *MAP3K1* expression in infiltrative GBM stem cells at the tumor periphery, and MAP3K1 knockdown significantly impaired invasion, proliferation, and stemness through reduced phosphorylation of c-jun at serine residues 63 and 73 [45]. Additionally, treatment with Nifuroxazide, a known STAT3 inhibitor, demonstrated anti-glioblastoma activity by enhancing tumor CD8+ T cell infiltration, reducing the expression of proteins such as ZEB2, N-cadherin, and MMP9, and inhibiting glioma cell invasion through suppressing the MAP3K1/JAK2/STAT3 pathway [109]. It is important to note that while MAP3K1 expression correlated with treatment outcomes, direct inhibition of MAP3K1 by nifuroxazide has not been biochemically demonstrated.

In hormone receptor (HR)-positive, human epidermal growth factor receptor 2 (HER2)-negative breast cancer, MAP3K1 knockdown significantly reduced migration and invasion both in vivo and in vitro by inhibiting JNK phosphorylation and NF-κB signaling, with MAP3K1 expression in clinical specimens strongly correlating with phospho-JNK levels and distant metastasis prediction [110]. Collectively, these studies indicate that MAP3K1 functions as a signaling node to regulate the metastatic cascade in cancer.

## 7. MAP3K1 in Inflammation and the Immune Microenvironment

While MAP3K1’s regulation of cell-intrinsic processes, including proliferation, survival, and motility, its influence extends beyond the cancer cell to the tumor microenvironment, particularly the immune component. MAP3K1’s multifaceted signaling outputs extend to immune cells, with profound consequences for anti-tumor immunity and immune evasion. In this section, we summarize MAP3K1’s role in regulating immune cell function and inflammatory responses, revealing mechanisms by which alterations in MAP3K1 reshape the tumor-immune interface.

**Figure 8:**
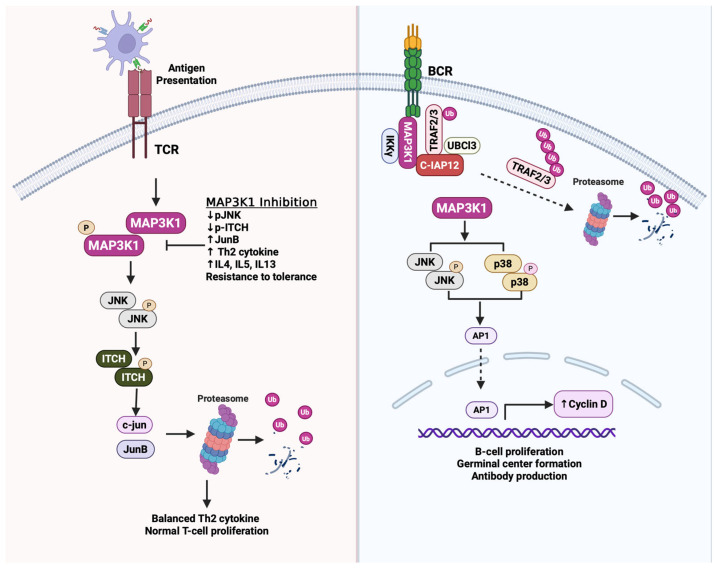
MAP3K1 modulates immune cells in a context-dependent manner. T Cell Regulation (**left**): Following T cell receptor (TCR) engagement, MAP3K1 activates JNK, which phosphorylates the E3 ubiquitin ligase Itch at serines 199, 222, and 232. Phospho-Itch ubiquitinates and degrades c-jun and JunB, negative regulators of IL-2 expression, thereby promoting T cell activation and proliferation. However, loss of MAP3K1 prevents JunB degradation, leading to excessive Th2 cytokine production (IL-4, IL-5, IL-13) and resistance to tolerance induction, creating an immunosuppressive tumor microenvironment. B Cell Activation (**right**): MAP3K1 is essential for CD40-mediated B cell responses. Upon CD40 ligation, a signaling complex containing TRAF proteins, c-IAP1/2, and MAP3K1 assembles at the receptor. Following TRAF3 degradation, the complex translocates to cytosol where MAP3K1 activates JNK and p38, promoting germinal center formation, antibody production, and B cell proliferation through AP-1 and cyclin D2 upregulation. Phosphorylation is shown as a circled “P” and ubiquitination is shown as a circled “Ub”. Created with BioRender.com.

MAP3K1 signaling plays a multifaceted, context-dependent role in the immune system, regulating T cell tolerance and proliferation, B cell activation and antibody production, and macrophage polarization (Figure 8). MAP3K1’s immune modulatory effects are associated with both anti-tumor immunity and immunosuppressive features in the tumor microenvironment, with context-dependent implications for cancer progression. Activation of the JNK pathway by MAP3K1, leading to T cell receptor engagement, is regulated by JNK-mediated phosphorylation of the E3 ubiquitin ligase Itch at specific residues (Ser199, Thr222, and Ser232) within its proline-rich region [111,112,113]. Phosphorylated Itch promotes the degradation of transcription factors such as c-jun and JunB, which are essential for cytokine expression and T-cell function [114,115]. Genetic deletion or mutations of MAP3K1, JNK1, and Itch lead to dysregulated accumulation of JunB and excessive Th2 cytokine production [116]. MAP3K1-knockout mice display expanded invariant natural killer T (iNKT) cell populations and enhanced interleukin (IL)-4 production, upregulating Th2-skewed immune responses. This indicates that MAP3K1 normally restrains iNKT cell expansion and Th2 polarization. Mechanistically, T cell-specific deletion of MAP3K1 impairs T cell proliferation through dysregulation of cyclin-dependent kinase inhibitor 1B (Cdkn1b). In wild-type T cells undergoing normal immune responses, MAP3K1 activates JNK signaling, which phosphorylates and promotes degradation of Cdkn1b, thereby releasing the brake on cell cycle progression and enabling proliferative expansion. Conversely, MAP3K1-deficient T cells accumulate Cdkn1b protein, resulting in impaired proliferation following T cell receptor (TCR) stimulation [117]. This MAP3K1-JNK-Cdkn1b regulatory axis is critical for physiological T cell expansion during antigen-specific immune responses and may similarly influence cancer cell proliferation in malignancies where this pathway is dysregulated.

Furthermore, MAP3K1 is essential for maintaining immune tolerance through the JNK-Itch signaling pathway. During tolerance induction, MAP3K1-activated JNK phosphorylates the E3 ubiquitin ligase Itch, which ubiquitinates and degrades the transcription factor JunB, thereby suppressing Th2 cytokine production and promoting T cell anergy. Consequently, T cells from MAP3K1-deficient, JNK1-deficient, or Itch-deficient mice fail to undergo normal tolerance induction, exhibiting constitutive JunB accumulation, unrestrained Th2 cytokine production, and spontaneous inflammatory disease resembling allergic airway inflammation [115,116]. These findings have important implications for cancer immunity and MAP3K1-targeted therapies. Effective anti-tumor responses require Th1-polarized immunity and cytotoxic T lymphocyte activation rather than Th2 responses [118,119]. suggesting that MAP3K1 deficiency-driven Th2 skewing could compromise tumor immune surveillance. Moreover, systemic MAP3K1 inhibition could trigger tolerance breakdown and autoimmune complications, as observed in MAP3K1-knockout mice. Therefore, context-dependent targeting strategies that preserve MAP3K1 function in immune cells may be necessary to avoid immune-related adverse events.

Beyond T cells, MAP3K1 regulates B cell-mediated immune responses. MAP3K1 is essential for cluster of differentiation-40 (CD40)-dependent activation of JNK and p38, germinal center formation, antibody production against thymus-dependent antigens, and B cell proliferation, through regulation of cyclin D2 expression and AP-1 transcription factor activation [39]. The molecular mechanism underlying MAP3K1 activation follows a two-stage signaling process at the CD40 receptor. In the first stage, CD40 engagement triggers rapid assembly of the IKK complex (IKKα, IKKβ, IKKγ/NEMO) at the receptor, leading to immediate IKK phosphorylation and NF-κB activation. In the second stage, a distinct signaling complex containing TRAFs, cellular inhibitor of apoptosis proteins (cIAP1/2), ubiquitin-conjugating enzyme 13 (UBC13), and MAP3K1 assembles and translocates from the receptor to the cytosol. This cytosolic translocation is essential for MAP3K1 activation and depends on cIAP1/2-mediated K48-linked ubiquitination and proteasomal degradation of TRAF3. This mechanism creates spatial and temporal separation between IKK/NF-κB activation (occurring rapidly at the receptor) and MAP3K1 activation (requiring delayed cytosolic translocation). The critical regulatory role of TRAF3 is underscored by frequent loss-of-function mutations in TRAF3 in multiple myeloma cell lines. Since TRAF3 normally suppresses MAP3K1 pathway activation, TRAF3 mutations result in constitutive MAP3K1 activation and dysregulated NF-κB signaling, thereby contributing to survival and proliferation of B-cell malignancies [120]. A *MAP3K1* variant (rs832582) associates with increased IL-1β, IL-6, and TNF-α production in acute respiratory distress syndrome, inducing a greater inflammatory response [121]. This pro-inflammatory phenotype parallels observations in MAP3K1-knockout mice, which exhibit spontaneous inflammatory disease and tolerance defects, suggesting a role for MAP3K1 in suppressing inflammation.

MAP3K1’s role in macrophage polarization is highly context dependent. MAP3K1 expression significantly influences macrophage polarization and inflammatory responses during infection after fracture fixation (IAFF), also known as osteosynthesis-associated infection (OAI). MiR-345-3p deficiency is associated with increased *MAP3K1* expression, accumulation of M1-type macrophages, and increased production of proinflammatory cytokines. miR-345-3p downregulates MAP3K1 and inhibits the phosphorylation of p38, JNK, ERK, and p65 in LPS-induced bone marrow-derived macrophages, thereby inhibiting M1 macrophage polarization, promoting the transition from M1 to M2, increasing the secretion of anti-inflammatory cytokines, and improving the inflammatory milieu in IAFF [122]. In hair follicle stem cells, MAP3K1 expression significantly altered MAPK and immune-related pathways, including IL-17 and TNF signaling, to regulate inflammation and cellular functions. Consequently, MAP3K1 overexpression promotes hair follicle stem cell proliferation by inhibiting apoptosis and facilitating cell cycle progression from S phase to G2 phase [123]. In systemic lupus erythematosus, elevated circulating miR-320b targets MAP3K1, reducing MAP3K1 expression and promoting pathogenic CD4+ T cell proliferation. Treatment with miR-320b antagomirs (synthetic miRNA inhibitors) in lupus mice restored MAP3K1 levels, suppressed CD4+ T cell proliferation, and ameliorated disease [124]. These findings indicate that the miR-320b/*MAP3K1* axis contributes to SLE pathogenesis and represents a potential therapeutic target. Additionally, MAP3K1 phosphorylates the transcriptional coactivator TORC1 (CRTC1) within its C-terminal activation domain (residues 431–650), promoting its nuclear translocation. Nuclear TORC1 is then recruited to cAMP response elements (CRE) at target gene promoters such as IL-8, facilitating IL-1-induced inflammatory gene expression [125].

In NSCLC, miR-770 is frequently downregulated. When tumor cells secrete exosomes containing miR-770, this miRNA targets *MAP3K1* in tumor-associated macrophages, reducing *MAP3K1* expression and decreasing JNK and ERK1/2 phosphorylation. This suppression inhibits M2 macrophage polarization, thereby reducing tumor cell invasion, migration, and epithelial–mesenchymal transition [108]. Loss of miR-770 in NSCLC removes this inhibition, permitting M2-mediated immune evasion. Conversely, in bone infection, MAP3K1 drives M1 macrophage polarization via NF-κB activation, promoting inflammatory tissue damage that is mitigated by miR-345-3p-mediated *MAP3K1* suppression [122]. These studies reflect differential engagement of downstream pathways (ERK/JNK versus NF-κB) and highlight the need for a context-specific understanding of MAP3K1 in macrophage-mediated diseases. However, the implications for cancer immunity are yet to be fully understood.

## 8. MAP3K1 Genetic Variations in Cancer

**Figure 9:**
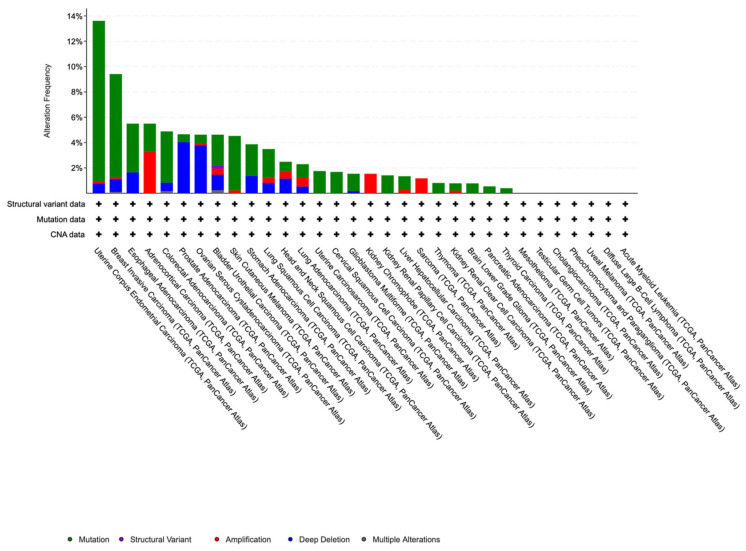
Landscape of MAP3K1 genetic alterations across human cancers. MAP3K1 alteration frequencies across 35 cancer types from The Cancer Genome Atlas. Bars show percentage of samples with MAP3K1 alterations, color-coded by alteration type: mutations (green), amplifications (red), deep deletions (blue), and multiple alterations (black). Highest frequencies occur in uterine corpus endometrial carcinoma (14%) and breast invasive carcinoma (10%), predominantly through mutations. Checkmarks indicate data availability: structural variants, mutations, and copy number alterations (CNA). Data accessed via cBioPortal (www.cbioportal.org) (accessed on 17 June 2025) [126,127].

The functional consequences of MAP3K1 signaling vary dramatically across cancer types, reflecting tissue-specific regulatory networks and mutational landscapes. The mutational landscape of MAP3K1 spans multiple cancer types, with MAP3K1 alterations across over 35 tumor types at frequencies ranging from <1% to 14% (Figure 9). The Cancer Genome Atlas (TCGA) data reveals remarkable heterogeneity across these tumor types, with the highest alteration frequencies observed in uterine corpus endometrial carcinoma (~14%) and breast invasive carcinoma (~10%), characterized by a predominance of loss-of-function mutations rather than amplifications or deletions, which is in contrast with predominantly missense mutations observed in other malignancies such as melanoma and lung cancer (Table 1).

**Figure 10:**
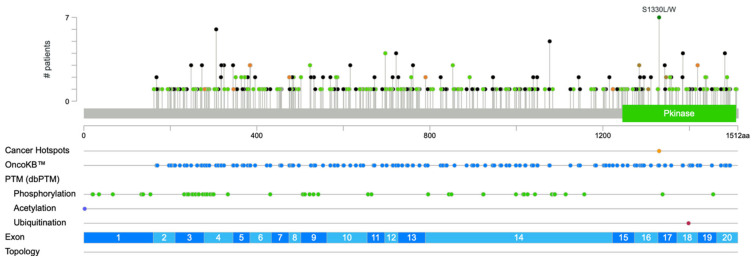
*MAP3K1* mutation landscape across the domain architecture. Distribution of *MAP3K1* mutations across the 1512-amino acid protein. Lollipops indicate mutation location and frequency, color-coded by type: missense (green), truncating (black), in-frame (brown). The kinase domain is highlighted in green (C-terminus). Annotation tracks show cancer hotspots, oncogenic predictions (OncoKB™), protein structure effects (PTM databases), and post-translational modification sites (phosphorylation, acetylation, ubiquitination). Notable hotspot: recurrent S1330L/W mutations in the PHD domain. Exon structure (20 exons) shown at bottom. Data accessed via cBioPortal (www.cbioportal.org) (accessed on 17 April 2025) [126,127].

Further analysis of mutation distribution from TCGA along the MAP3K1 protein sequence demonstrates non-random clustering in functionally critical domains (Figure 10; Table 2). Several notable recurrent hotspot mutations appear across multiple cancer types, including S1330L in the kinase domain (occurring in glioma, ovarian, stomach, and colorectal cancers), R306H in the SWIM domain (appearing in endometrial, colorectal, and prostate cancers), and X475_splice mutations in the PHD/RING domain (found in lung and head and neck cancers).

These mutations may disrupt kinase activity, protein–protein interactions, and E3 ligase function. Despite numerous studies reporting specific mutations and their frequencies, the lack of validated functional effects for many of these genetic variants limits translational studies. For instance, the MAP3K1 rs889312 SNP increases transcriptional activity and exhibits a protective effect against pancreatic cancer, whereas it typically increases breast cancer risk; however, its functional relevance remains to be characterized [154]. Importantly, cataloging genetic alterations represents the first step toward clinical translation. Functional validation of the MAP3K1 alterations will provide actionable information and enhance clinical significance. The disconnect between genetic identification and functional characterization is a knowledge gap that must be addressed to enable the translation of *MAP3K1* mutational studies into clinically actionable biomarkers.

## 9. Biomarkers, Prognosis, and Outcome

Expression levels and mutational status of MAP3K1 have been identified as both prognostic indicators of clinical outcomes and potential predictive biomarkers of treatment response observed across multiple cancer types (Table 3). The clinical significance of MAP3K1 alterations is context-dependent, varying by cancer type, co-occurring genetic alterations, and therapeutic regimen. While MAP3K1 is generally associated with poor outcomes in some solid tumors through pro-survival signaling, it paradoxically predicts favorable outcomes when MAP3K1-mediated apoptotic signaling is preserved or when loss-of-function mutations prevent compensatory pathway activation. This section examines MAP3K1 as a clinically actionable biomarker, highlighting contexts in which *MAP3K1* status informs prognosis and treatment selection.

In solid tumors, including glioblastoma, bladder cancer, and hormone receptor-positive breast cancer, elevated MAP3K1 expression is associated with poor prognosis, enhanced metastatic potential, and treatment resistance [110,155,156]. These associations likely reflect MAP3K1’s role in activating pro-survival NF-κB and ERK signaling and in regulating DNA damage responses that enable tolerance to genotoxic stress. In glioblastoma, combined elevation of TRIB2 and MAP3K1 expression correlates with poor prognosis and resistance to temozolomide chemotherapy, suggesting MAP3K1 may contribute to therapy resistance through activation of pro-survival signaling pathways [155]. In cholangiocarcinoma, *MAP3K1* expression negatively correlates with rigosertib sensitivity, a polo-like kinase 1 (PLK1) inhibitor was subsequently found to bind to MAP3K1 directly, which shows drug-protein interactions using structural and biochemical studies [157]. Conversely, in acute myeloid leukemia, higher MAP3K1 expression significantly correlates with better prognosis and improved survival [158,159], highlighting MAP3K1’s dual nature. This divergent prognostic value could be attributed to the context-specific effects on pro-survival and pro-apoptotic outputs. In AML with intact apoptotic machinery, MAP3K1-mediated JNK activation enhances chemotherapy-induced apoptosis [28,59,160], whereas in solid tumors, compromised apoptotic pathways and pro-survival signaling predominate. In hepatocellular carcinoma, *MAP3K1* mutations detected in circulating tumor (ct) DNA are associated with early recurrence and systemic therapy resistance [146], indicating that ctDNA-based MAP3K1 monitoring could be a useful tool for predicting recurrence risk.

However, the prognostic value of MAP3K1 in hepatocellular carcinoma appears treatment-dependent, with high MAP3K1 expression predicting superior survival specifically after sorafenib treatment [161]. This association may reflect sorafenib’s off-target inhibition of MAP3K1, as revealed by KiNativ profiling, where patients with higher baseline MAP3K1 expression may derive greater benefit from its pharmacological inhibition [162]. Additional associations with genetic variants have been reported in NSCLC and other cancers (Table 3), although the functional consequences of many of these variants remain to be characterized.

In breast cancer, *MAP3K1* mutations frequently co-occur with phosphatidylinositol-4,5-bisphosphate 3-kinase catalytic subunit alpha (*PIK3CA*) mutations, and this combined profile is associated with particularly favorable survival outcomes in retrospective cohorts [9,163]. A recent large-scale validation study of 1780 luminal breast cancer patients from the TCGA and METABRIC cohorts has reinforced the favorable prognostic implications of *MAP3K1* mutations. The study used three clinically validated risk-stratification tools (Oncotype DX, MammaPrint, and PAM50-ROR) and found that *MAP3K1* mutations were significantly enriched in low-risk groups. Notably, co-mutations of *PIK3CA-MAP3K1* and *KMT2C-MAP3K1* were identified in the low-risk group, whereas enrichment of *TP53*, *RB1*, and *PTPN22* mutations, along with co-mutations in *TP53-PIK3CA* and *TP53-KMT2C*, were identified in the high-risk group. Intriguingly, although *PIK3CA* mutations are generally associated with favorable outcomes, *PIK3CA*-mutant tumors in the high-risk group showed worse recurrence and survival outcomes, suggesting that the prognostic implications of *MAP3K1* and *PIK3CA* co-mutations are context-dependent and influenced by the broader mutational landscape [164]. Additionally, analysis of 1795 HR+/HER2-negative breast cancers from the BIG 1-98 and SOFT trials subdivided by HER2 expression level revealed *MAP3K1* mutations as the primary genomic distinction between HER2-zero tumors (IHC 0) and HER2-low tumors (IHC 1+ or 2+/ISH-negative), occurring in 19% versus 5% (BIG 1-98) and 11% versus 6% (SOFT), respectively. Although HER2-low and HER2-zero groups display similar clinical outcomes, *MAP3K1* mutation status may represent a more biologically meaningful classifier, potentially reflecting distinct evolutionary pathways in ER+ breast cancer development [165].

In contrast to its tumor-suppressive role in breast cancer, MAP3K1 functions as an oncogene in melanoma [88,89], where alterations co-occur with BRAF mutations and contribute to amplification of oncogenic ERK signaling. This divergence reflects distinct genomic ecosystems: MAP3K1 loss is tolerated in PIK3CA-mutant breast cancers with alternative PI3K-AKT activation, whereas MAP3K1 promotes MAPK pathway hyperactivation in BRAF-mutant melanomas. These opposing functions—tumor suppressor in hormone receptor-positive breast cancer versus oncogene in melanoma—illustrate MAP3K1’s remarkable context-dependency, shaped by tissue-specific signaling networks and co-occurring genetic alterations.

*MAP3K1* mutational status predicts response to both chemotherapy and targeted therapies. *MAP3K1* mutational status is associated with sensitivity to MEK inhibitors across multiple cancer models [8]. Mechanistically, sustained MEK inhibition triggers compensatory mechanisms through JNK-c-jun activation, which mediates survival and therapeutic resistance. Loss-of-function *MAP3K1* mutations prevent this compensatory feedback and eliminate a resistance mechanism. This suggests MAP3K1-mutant cancers, which are frequent in breast, prostate, and colon, may be exceptionally responsive to MEK inhibitors. Consistently, in a small cohort of biliary tract cancer patients who responded to binimetinib (MEK inhibitor) plus chemotherapy, a *MAP3K1* mutation was identified [151]. Conversely, in *PIK3CA*-mutant breast cancer, *MAP3K1* loss-of-function mutations create PI3K inhibitor resistance. MAP3K1 depletion decreases JNK signaling, thereby increasing Insulin receptor substrate 1 (IRS1) stability and enhancing PI3K pathway activation, a predicted resistance mechanism that enables cells to bypass AKT inhibition [9]. This suggests that *PIK3CA*-mutant patients with intact *MAP3K1* are likely to respond better to PI3K-targeted therapies than those with co-occurring *MAP3K1* mutations or loss. Computational predictions suggest *MAP3K1* mutations may be deleterious in colorectal cancer, though experimental validation of these observations has yet to be reported [166]. A MAP3K1 missense variant identified in breast cancer disrupts protein function. It acts as a driver mutation [132], whereas *MAP3K1* mutations in invasive breast carcinoma correlate with lower tumor mutational burden [167], though the functional link underlying this association is unclear. Beyond MEK and PI3K pathway inhibitors, genome-wide CRISPR screens have identified synthetic lethal partners of *MAP3K1*, with WEE1 kinase (WEE1) showing the strongest synthetic lethality. *MAP3K1*-mutant cells demonstrate increased sensitivity to the WEE1 inhibitor AZD1775; a Phase II clinical trial (NCT04158336) reported a 38% response rate, validating WEE1 inhibition as a clinically actionable strategy in MAP3K1-mutant cancers [168,169,170]. These observations underscore the broader challenge of stratifying oncogenic *MAP3K1* variants from passenger mutations.

For chemotherapy, the predictive value of *MAP3K1* varies by agent and context. High MAP3K1 expression is associated with resistance to temozolomide (TMZ) in glioblastoma and to platinum agents in bladder cancer [155,156], and correlates with activation of survival pathways and DNA damage tolerance mechanisms. In glioblastoma stem cells, the lncRNA nuclear paraspeckle assembly transcript 1 (NEAT1) promotes temozolomide resistance by functioning as a competing endogenous RNA that sequesters let-7g-5p, thereby upregulating MAP3K1 expression [171]. MAP3K1 also drives hypoxia-induced chemoresistance by upregulating P-glycoprotein (P-gp) via the JNK-HIF-1α pathway [172]. Paradoxically, in hematologic malignancies, elevated MAP3K1 expression sensitizes cells to microtubule-targeting agents by facilitating BCL-2 phosphorylation and PARP cleavage-mediated apoptosis [173,174]. Genome-wide analysis of >11,000 cancers revealed that *MAP3K1* can acquire loss-of-function mutations during chemotherapy, and high-impact *MAP3K1* mutations (nonsense, frameshift, and splice-site alterations) increased from 9% to 23% in antiestrogen-treated breast cancer patients [175]. Mechanistically, *MAP3K1* loss removes restraint on PI3K/AKT signaling, particularly in *PIK3CA*-mutant backgrounds, conferring resistance. This creates a paradox: *MAP3K1* loss shifts from a favorable prognostic marker in untreated tumors to a resistance mechanism under therapeutic pressure, suggesting that *MAP3K1* loss confers vulnerability to PI3K/AKT pathway inhibitors. Together, these studies point to a highly context-dependent prognostic and predictive value of MAP3K1 alterations, which varies by cancer type, co-occurring mutations, and treatment regimen (Table 3).

**Table 3:** MAP3K1 Biomarker Associations Across Cancer Types.

Cancer Type.	*MAP3K1* Status	Biomarker Type	Clinical Association	Treatment Context	Proposed Mechanism	References
POOR PROGNOSIS						
Glioblastoma	High expression	Prognostic and Predictive	Poor prognosis; TMZ resistance	TMZ treatment	Activates survival signaling (NF-κB), regulates DNA damage response	[155]
Bladder cancer	High expression	Prognostic and Predictive	Predicts recurrence; Cisplatin resistance	Cisplatin	Enhanced survival signaling	[156]
Breast (HR+/HER2-)	High expression	Prognostic	Poor prognosis; ↑ metastasis	General	Enhanced proliferation, migration, invasion via JNK/NF-κB	[110]
Cholangiocarcinoma	High expression	Predictive	Rigosertib resistance	Rigosertib	Direct drug-protein interaction	[157]
Hepatocellular carcinoma	*MAP3K1* mutations (ctDNA)	Prognostic	Early recurrence; Therapy resistance	Systemic therapies	Loss of tumor suppressor function	[146]
NSCLC	Genetic variants (SNPs)	Prognostic	Poor overall survival	Platinum + taxane	Unclear; requires functional validation	[176]
Squamous cell carcinoma	Gene amplification	Predictive	PDT resistance	Photodynamic therapy	ERK1/2 activation → proliferation	[177]
Triple-negative breast cancer (TNBC)	High expression (epirubicin-resistant)	Predictive	Epirubicin resistance	Epirubicin	ERK pathway activation	[178]
Breast (*PIK3CA*-mutant)	*MAP3K1* loss/mutation	Predictive	PI3K inhibitor resistance	PI3K pathway inhibitors	↓ JNK → ↑ IRS1 → ↑ PI3K activation	[9]
Multiple cancers	High expression (NEAT1-mediated)	Predictive	Chemoresistance	Various chemotherapies	NEAT1/let-7g-5p/MAP3K1 axis	[171]
FAVORABLE PROGNOSIS						
Acute myeloid leukemia	High expression	Prognostic	Better prognosis; Improved survival	General	Preserved apoptotic signaling capacity	[158,159]
Breast cancer (ER+)	*MAP3K1* + *PIK3CA* mutations	Prognostic	Favorable survival	General	Prevents compensatory PI3K activation	[9,163]
Hepatocellular carcinoma	High expression	Prognostic	Superior survival	Sorafenib treatment	Treatment-specific mechanism (unclear)	[179]
Multiple cancer models (Breast, Colorectal)	*MAP3K1* mutations (LOF)	Predictive	MEK inhibitor sensitivity	MEK inhibitors	Prevents compensatory JNK-c-jun activation	[8]
Biliary tract cancer	*MAP3K1* mutations	Predictive	Treatment response	Binimetinib + gem/cis	Prevents feedback resistance	[151]
Leukemia	High expression	Predictive	Microtubule-targeting agent sensitivity	Taxanes, vinca alkaloids	Facilitates BCL-2 phosphorylation/cleavage	[173,174]
Prostate cancer	Death receptor-induced MAP3K1	Predictive	Chemosensitization	Paclitaxel + platinum	MAP3K1 → JNK → apoptosis	[180]

"↓" denotes downregulation, whereas "↑ " denotes upregulation.

Together, the above studies establish that *MAP3K1* status, whether measured by expression level or mutational status, correlates with both prognosis and treatment response across multiple cancer types. These associations suggest that MAP3K1 is a clinically actionable target. Still, they also reveal complexity: the same *MAP3K1* alteration can predict favorable outcomes in one therapeutic context and unfavorable outcomes in another, reflecting context-dependent signaling outputs we have highlighted throughout this review. This complexity suggests the need for additional studies and thoughtful therapeutic strategies that account for MAP3K1’s dual nature.

## 10. Targeting MAP3K1 for Cancer Therapeutics

Understanding MAP3K1’s context-dependent biology has direct implications for therapeutic strategies and biomarker development. In this section, we examine approaches to therapeutically exploit *MAP3K1* status in cancer. These range from direct pharmacological inhibition to the use of *MAP3K1* mutations for treatment selection. Rational MAP3K1-directed therapeutic development requires matching the strategy to functional-context considerations. The diverse, context-dependent functions of MAP3K1 in cancer biology offer both opportunities and challenges for therapeutic targeting. In contexts where MAP3K1 is associated with pro-survival signaling, chemoresistance, and metastasis, direct MAP3K1 inhibition or indirect targeting through downstream pathways would be a viable therapeutic strategy. Conversely, in contexts where MAP3K1 mediates pro-apoptotic signaling or acts as a tumor suppressor, therapeutic approaches should preserve MAP3K1 function. *MAP3K1* mutational status can also guide selection among existing therapies, particularly MEK and PI3K pathway inhibitors.

Direct pharmacological inhibition of MAP3K1 remains limited, with IKAM-1 (IKKβ Activation Modulator-1) and compound 51–106 as the only reported MAP3K1-selective small-molecule inhibitors to date. These compounds effectively prevent MAP3K1-mediated phosphorylation of IKKβ at serines 177 and 181, thereby blocking NF-κB activation, a key pathway by which MAP3K1 promotes cancer cell survival, proliferation, and metastasis. In preclinical pancreatic cancer models, both IKAM-1 and 51–106 demonstrate significant anti-tumor activity, inhibiting tumor growth, metastatic dissemination, and angiogenesis, providing proof of concept that MAP3K1 is a druggable target [69,96]. Given the multi-domain, multi-functional nature of MAP3K1, the targeted protein degradation approach, such as proteolysis targeting chimeras (PROTACS), could reveal the biological consequences of MAP3K1 loss beyond kinase inhibition.

Due to the limited availability of pharmacological inhibitors, most validation studies have relied on genetic approaches, including shRNA, siRNA, CRISPR, and microRNA-based modulation. These genetic approaches have been instrumental in validating MAP3K1 as a therapeutic target and in defining contexts in which inhibition provides benefit. For example, in HR-positive, HER2-negative breast cancer, shRNA-based MAP3K1 depletion induced G2/M cell cycle arrest, reduced cyclin B1 expression, triggered apoptosis, and sensitized cells to doxorubicin, docetaxel, and tamoxifen [110]. An artificial microRNA targeting MAP3K1 similarly suppressed tumor growth and metastasis in breast cancer models [181]. Multiple microRNAs have been reported to regulate MAP3K1 across various cancer types with context-dependent outcomes (Table 4). These studies demonstrate MAP3K1’s context-dependent role in cancer, with MAP3K1 inhibition suppressing proliferation, reducing metastatic capacity, overcoming chemoresistance, and inducing cell cycle arrest in specific contexts, validating MAP3K1 as a potential therapeutic target.

A recent comprehensive multi-level profiling across 405 breast cancer patients has revealed coordinated dysregulation of multiple miRNAs targeting *MAP3K1* across all molecular subtypes. Five miRNAs (miR-21-3p, miR-23c, miR-27a-3p, miR-205-3p, and miR-300) exhibited inverse expression patterns relative to *MAP3K1*, with the most pronounced miRNA upregulation observed in TNBC. In this context, *MAP3K1* downregulation impairs MAPK-mediated apoptotic signaling, weakens cellular stress responses, and suppresses intrinsic apoptotic pathways [187]. Nifuroxazide, an FDA-approved antidiarrheal drug repurposed for cancer therapy, targets the JAK2/STAT3 pathway in glioblastoma, and treatment response has been shown to correlate with MAP3K1 expression. Nifuroxazide treatment reduces expression of invasion-associated proteins, including ZEB2, N-cadherin, and MMP9, ultimately suppressing glioblastoma invasive capacity. Furthermore, nifuroxazide, when combined with PD-L1 inhibitors, prolongs survival and enhances CD8+ T cell infiltration in preclinical glioblastoma models. This synergy may reflect nifuroxazide’s anti-tumor effects through MAP3K1 inhibition in glioblastoma cells, thereby reducing invasion-associated proteins and enhancing T cell-mediated immunity through PD-L1 blockade [109]. Although MAP3K1 plays a significant role in cancer metastasis, the precise impact of MAP3K1 inhibition on the glioblastoma immune microenvironment and its effect on the nifuroxazide mechanism remain unclear.

MAP3K1 expression has been associated with resistance to multiple chemotherapeutic agents by activating pro-survival pathways, providing a rationale for combining chemotherapy with MAP3K1 inhibition. For example, in glioblastoma, elevated MAP3K1 expression is associated with temozolomide resistance, and MAP3K1 inhibition sensitizes cells to TMZ by disrupting NF-κB survival signaling and DNA damage responses [155]. In TNBC, epirubicin-resistant cells show enrichment for MAP3K1 and increased ERK phosphorylation, suggesting that MAP3K1-ERK signaling contributes to anthracycline resistance, even in cancers not initially ERK-dependent [178]. This may expand the population of patients who may benefit from combining chemotherapy with MAP3K1 or ERK pathway inhibition. Caspase-3-mediated cleavage of MAP3K1 acts as a molecular switch that favors apoptotic signaling; MAP3K1 functions as a tumor suppressor, and several chemotherapeutic agents exploit this mechanism. For example, in prostate cancer cells, paclitaxel induces FADD phosphorylation at Ser194 through JNK activation, which subsequently upregulates MAP3K1 expression. This paclitaxel-mediated upregulation of MAP3K1 primes cells for enhanced apoptosis: when cells are subsequently treated with etoposide or cisplatin, the elevated MAP3K1 levels amplify JNK activation and significantly enhance apoptotic responses. Importantly, blocking FADD phosphorylation using an S194A mutant prevents MAP3K1 upregulation and abolishes the synergistic apoptotic effects, demonstrating that FADD-dependent MAP3K1 induction is essential for paclitaxel-mediated chemosensitization [180]. MAP3K1 also mediates death receptor-induced apoptosis in response to DNA damage. Etoposide treatment activates MAP3K1, which in turn activates NF-κB, promoting its nuclear translocation and binding to the DR4 promoter to drive death receptor 4 (DR4) transcription. This MAP3K1-dependent DR4 upregulation sensitizes cells to TRAIL-induced apoptosis, and expression of kinase-dead MAP3K1 abolishes both DR4 induction and TRAIL sensitization [80,81]. In these contexts, MAP3K1 inhibition would be counterproductive, potentially reducing chemotherapy efficacy by eliminating pro-apoptotic signaling. MAP3K1 loss drives metabolic reprogramming through HIF-1α stabilization and Mammalian target of rapamycin (mTOR) activation, resulting in glutamine addiction, with upregulation of glutaminase 1 and therapeutic vulnerability to the glutaminase inhibitor CB-839 [188,189], representing an alternative metabolic-targeting approach in MAP3K1-deficient tumors. On the other hand, high MAP3K1 expression is correlated with enhanced sorafenib response in hepatocellular carcinoma [179], whereas in Hedgehog-driven medulloblastoma, MAP3K1 functions as a tumor suppressor, making inhibition unfavorable [77]. Therefore, clinical translation faces several critical challenges, given that MAP3K1’s dual oncogenic and tumor-suppressor roles necessitate a functional context and the use of robust biomarkers beyond mutation detection.

## 11. Conclusions

This review summarizes progress over the past decade in understanding MAP3K1’s role in cancer biology. During this time, our understanding of this unique dual-function kinase has undergone a fundamental transformation. What was once viewed primarily as a stress-activated kinase within the MAPK cascade is now recognized as a multifaceted signaling hub whose functions extend far beyond canonical pathway activation to encompass E3 ubiquitin ligase activity, direct transcriptional co-regulation, control of microtubule dynamics, and modulation of the immune microenvironment. Given several studies establishing MAP3K1 as an essential protein in cancer biology, the challenge moving forward lies in understanding the rules that govern when MAP3K1 acts as a friend or foe and in translating this knowledge into clinical strategies.

**Figure 11:**
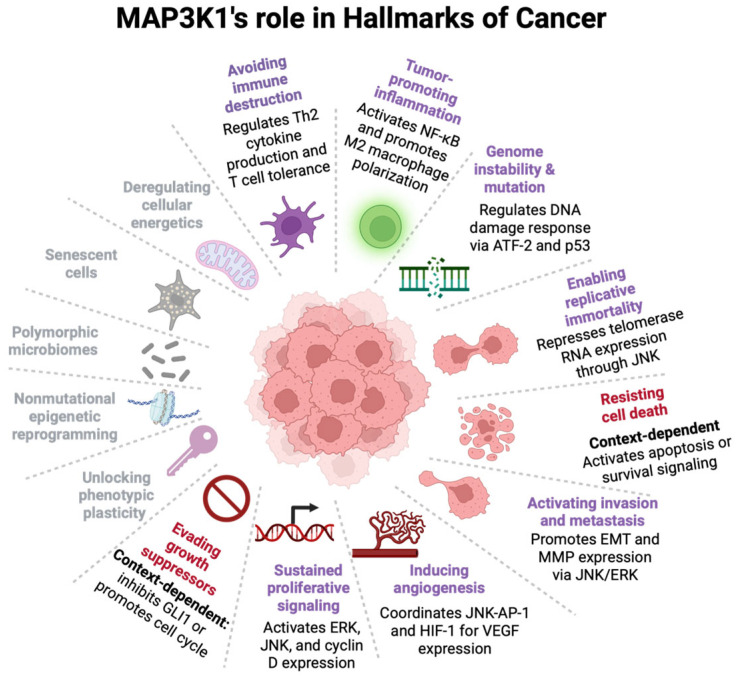
MAP3K1 contributes to nine of fourteen cancer hallmarks through context-dependent mechanisms: Tumor-promoting roles (purple) include inflammation via NF-κB and macrophage polarization; DNA damage response regulation through ATF-2/p53; telomerase repression via JNK; invasion/metastasis through EMT and MMP expression (JNK/ERK); angiogenesis via JNK-AP-1/HIF-1-mediated VEGF; and sustained proliferation through ERK/JNK and cyclin D. Context-dependent tumor-suppressive or tumor-promoting roles (red): context-dependent apoptosis activation. Potential involvement (gray): growth suppressor evasion, immune destruction avoidance, metabolic reprogramming, senescence, microbiomes, epigenetics, and phenotypic plasticity. Hallmarks framework from Hanahan and Weinberg [127]. Created with BioRender.com.

MAP3K1’s context-dependent function reflects its unique position as a molecular switch that can elicit opposed cellular outcomes in response to various stimuli. MAP3K1 is a unique, multifunctional protein within the MAPK cascade, with dual enzymatic activities and a complex domain structure that enables remarkably context-dependent functions across cancer types. The multidomain architecture of MAP3K1 provides the molecular basis for its diverse signaling outputs and context-dependent effects. At the pathway level, MAP3K1 simultaneously activates pro-survival (NF-κB, ERK) and pro-apoptotic (JNK, p38) cascades, with caspase-3 cleavage at DTVD-878 serving as one of the switches, generating a 91 kDa fragment that preferentially activates pro-apoptotic pathways. At the expression level, high MAP3K1 is associated with poor prognosis in some cancers, such as lung cancer, where it drives survival signaling and chemoresistance; in acute myeloid leukemia, it mediates chemotherapy-induced apoptosis. This context-dependence extends across cancer hallmarks: our analysis reveals that MAP3K1 influences at least nine of the fourteen established hallmarks (Figure 11), positioning MAP3K1 as a cancer-associated protein with context-dependent outcomes. By activating NF-κB, MAP3K1 drives a tumor-promoting microenvironment. Regulation of ATF-2 and p53 by MAP3K1 connects it to genomic instability and DNA damage response pathways. JNK-mediated repression of telomerase RNA by MAP3K1 contributes to replicative immortality. Through the JNK and ERK signaling, MAP3K1 promotes epithelial–mesenchymal transition and matrix metalloprotease expression, activating invasion and metastasis. The coordination of JNK-AP-1 and HIF-1 pathways for VEGF expression establishes a role for MAP3K1 in inducing angiogenesis. By activating ERK, MAP3K1 upregulates cyclin D expression to sustain proliferative signaling. The unique combination of dual enzymatic activities (kinase and E3 ligase) of MAP3K1, its integration into four major signaling pathways (NF-κB, ERK, JNK, p38), and its extensive protein–protein interaction network position it to wear multiple hats in the hallmarks of cancer (Figure 11).

Despite substantial progress, significant knowledge gaps limit our ability to exploit MAP3K1 therapeutically in cancer. The molecular determinants governing the context-dependent behavior of MAP3K1 require a systematic, comprehensive investigation using integrative genomic, proteomic, and functional approaches. The interplay between the dual enzymatic activities (kinase and E3 ligase) and the complete substrate repertoire of its understudied E3 ligase function remains poorly understood. Critical research priorities include: (a) functional characterization of *MAP3K1* variants using high-throughput approaches such as deep mutational scanning combined with structural biology to determine their effects on kinase, E3 ligase, and scaffolding activities; (b) validation of E3 ligase substrates beyond current speculative predictions; (c) development of selective pharmacological tools to independently target MAP3K1’s kinase or E3 ligase functions for precise modulation of signaling outputs; and (d) prospective validation of connections between *MAP3K1* mutations and clinically relevant phenotypes to identify potential biomarkers and therapeutic resistance mechanisms. The prognostic and predictive value of *MAP3K1* status may be complicated by temporal heterogeneity, as MAP3K1 alterations can be acquired during disease progression and recurrence, and snapshots of genetic profiling may not capture the full spectrum of MAP3K1 alterations relevant for clinical utility [190]. Progress has been hindered by methodological challenges, including difficulty experimentally distinguishing among MAP3K1’s enzymatic activities; limited availability of well-validated antibodies that detect endogenous MAP3K1 forms and modifications; the limited number of MAP3K1 inhibitors; and the limited number of tissue- and genetic background-specific models, creating a disconnect between genetic data and functional understanding. A comprehensive investigation of the role of MAP3K1 in the tumor microenvironment, particularly whether blocking tumor-mediated suppression enhances immunotherapy and how *MAP3K1* mutations alter the immune landscape, could reveal new opportunities for combination therapy. Leveraging emerging technologies, including single-cell multi-omics, spatial transcriptomics/proteomics, CRISPR-based editing, patient-derived organoids, and intravital imaging, would provide unprecedented insights into MAP3K1 biology in native contexts.

A retrospective analysis of MEK inhibitor outcomes in MAP3K1-mutant versus wild-type cancers across tumor types is a rational approach to establish clinical utility. Given the mechanistic basis where *MAP3K1* mutations drive constitutive ERK activation, such studies could validate *MAP3K1* mutational status as a predictive biomarker to guide patient selection for MEK inhibitor therapy. Furthermore, *MAP3K1* status can inform patient selection for PI3K pathway inhibitors, with *MAP3K1* loss in *PIK3CA*-mutant breast cancer predicting resistance and suggesting either exclusion from single-agent trials or enrollment in combination therapy cohorts. MAP3K1 expression could guide combination chemotherapy strategies in specific contexts. For example, given promising preclinical synergy, combining TMZ with MAP3K1 inhibitors in high-MAP3K1 glioblastoma, or testing the role of MAP3K1 inhibition plus PD-L1 inhibitors on tumor progression, merits investigation. Longer-term clinical translation requires investment in the development of pharmacological modulators of MAP3K1 through multiple approaches, including ATP-competitive, allosteric, E3-ligase-selective, covalent binders and targeted protein degraders.

In conclusion, MAP3K1 is a critical regulator of cancer cell survival and apoptosis. The genetic, molecular, and clinical evidence establishing its importance in cancer biology is now overwhelming: it ranks among the most frequently altered genes across cancer types, influences multiple aspects of cancer hallmarks, predicts treatment responses, and shows compelling preclinical evidence for therapeutic targeting. This gap between knowledge and application presents a unique opportunity to study a protein with multi-domain function and context-dependent tumor-promoting and tumor-suppressing activities. The convergence of mechanistic insights, genomic data, and early clinical evidence positions MAP3K1 at a pivotal moment for translational oncology. As a molecular switch with context-dependent tumor-promoting and tumor-suppressing activities, MAP3K1 offers a paradigm for understanding how signaling networks generate diverse outputs, insights applicable to many other pleiotropic cancer genes.

## Figures and Tables

**Table 1:** MAP3K1 genetic alteration across cancer types.

Cancer Type	Mutation	Common Alterations	Functional Impact	Clinical Implications	References
Breast Cancer	6–14% overall; higher in ER+ and grade 1 tumors	Truncating mutations (59–74%); missense mutations; rs889312 polymorphism	Loss-of-function; disrupts MAPK/JNK pathway	Favorable prognosis in hormone receptor-positive (HR+) cancers; enriched in luminal A subtype	[9,128,129,130,131,132,133,134,135,136,137,138]
Hormone Receptor-Positive Breast Cancer	8% of HR+ cases	rs889312 (C/C vs. C/A+A/A)	Reduces sensitivity to PI3K pathway inhibitors	Poor overall survival in premenopausal women; potential biomarker	[139,140,141]
Diffuse-type Gastric Cancer	rs889312 AC genotype: 49.7%	rs889312 and rs252902 polymorphisms lncRNA MAP3K1-2 upregulation	Alters protein expression; affects ER, NF-κB signaling promotes proliferation and invasion	Poorer survival (HR = 1.32); independent prognostic factor Increased risk in men; poor prognosis with high expression	[142,143,144]
Hepatocellular Carcinoma	Among the top 5 mutated genes in ctDNA	Missense mutations	Associated with resistance and recurrence	Predicts early recurrence; worse progression-free survival	[145,146]
Melanoma	Recurrent in desmoplastic subtype	Insertional mutations in introns 9–10	Gain-of-function; activates ERK signaling	Mutually exclusive with BRAF V600E; potential therapeutic target	[88,147]
Colorectal Cancer	Not specified	rs702689 polymorphism	May influence metastasis	Associated with lymph node metastasis risk	[148]
Leukemia (MPAL)	11% of cases	Various mutations in MAP3K families (MAP3K1 and MAP3K6)	Alter proliferation and differentiation	Recurrent mutations in MAP3K family	[149]
Sarcoma	Few mutations	Not specified	MAPK signaling alteration, response to targeted treatments	Higher frequency in Chinese vs. Western populations	[150]
Biliary Tract Cancer	Mutations in the MAPK pathway, including MAP3K1	Variants in MAP3K1	Affects MAPK pathway signaling	Found in binimetinib responders	[151]
Nasopharyngeal Carcinoma	10% in NPAC subtype	Not specified	Not specified	Among the top 5 mutated cancer-related genes	[152]
Overall Cancer Prognosis	Varies by cancer type	rs889312 polymorphism	Affects cell growth, proliferation, differentiation pathways	Associated with poor survival in some cancers, protective in others	[130,153]

**Table 2:** Distribution of *MAP3K1* Mutations by Cancer Type and Protein Domain.

Cancer Type	Common Mutation Types	Notable Recurrent Mutations	Affected Protein Domains	Frequency
Breast Invasive Carcinoma	Frameshift, Nonsense	R273Sfs*27, R763Cfs*35, L319Tfs*7, Q1048*	Kinase domain (C-terminal), PHD/RING domain (N-terminal), SWIM domain	High (>50 cases)
Uterine Endometrial Carcinoma	Frameshift, Nonsense	E722 *, E1343 *, G617Efs*39, N1079Ifs*3	Kinase domain (C-terminal), Scaffold region	High (>40 cases)
Colorectal Adenocarcinoma	Nonsense, Missense	S1330L, R208 *, S423 *	Kinase domain (C-terminal), SWIM domain (N-terminal)	Moderate
Stomach Adenocarcinoma	Nonsense, Frameshift	S1330L, R248 *, Q1207 *	Kinase domain (C-terminal), SWIM domain (N-terminal)	Moderate
Lung Cancer	Nonsense, Splice site	Q345 *, X475_splice	SWIM domain, PHD/RING domain	Low
Melanoma	Frameshift, Missense	T946Nfs *57, X211_splice	Scaffold region, Kinase domain	Low
Glioma	Missense, Frameshift	S1330L, N749Mfs *5	Kinase domain (C-terminal)	Low
Bladder Urothelial Carcinoma	Nonsense	S640 *, R1482 *, Q977 *	Scaffold region, Kinase domain	Low
Thyroid Cancer	Nonsense	S1049 *, G1311 *	Kinase domain (C-terminal)	Very low

The * Denotes a stop codon. Data generated using cBioPortal (www.cbioportal.org) [126,127].

**Table 4:** Distribution of MAP3K1-targeting miRNAs by cancer type and functional outcome.

miRNA	Cancer Type	Mechanism of Action	Functional Outcomes	Reference
miR-375	Colorectal carcinoma	Direct binding to MAP3K1 3'UTR	Decreased cell growth, induced apoptosis	[166]
miR-196b	Choriocarcinoma	Direct binding to MAP3K1 3'UTR	Suppressed cell proliferation, migration, and invasion	[182]
miR-206	Hepatocellular carcinoma (HCC)	Direct binding to MAP3K1 3'UTR	Suppressed MAP3K1 expression (mRNA and protein levels), inhibited HCC cell proliferation, migration, and invasion	[179]
miR-770	Non-small cell lung cancer (NSCLC)	Targeting MAP3K1	Inhibition of M2 macrophage polarization, suppressed NSCLC cell invasion	[108]
miR-23b	Not specified	Targeting MAP3K1 and other pro-metastatic genes	Inhibited tumor growth, invasion, and angiogenesis	[183]
miR-145-5p	Non-small cell lung cancer (NSCLC)	Direct binding to MAP3K1 3'UTR	Increased E-cadherin levels, suppressed vimentin, reduced MAP3K1 mRNA and protein levels, inactivated JNK signaling pathway	[184]
miR-451	Esophageal carcinoma	Direct targeting of MAP3K1	Inhibited cell proliferation and tumor growth	[185]
miR-203	Esophageal cancer	Targeting MAP3K1	Decreased MAP3K1 expression, inhibited cell proliferation and invasion, induced apoptosis	[186]

## Data Availability

No new data were created or analyzed in this study.
